# Adequacy and tolerance to ass's milk in an Italian cohort of children with cow's milk allergy

**DOI:** 10.1186/1824-7288-35-19

**Published:** 2009-07-09

**Authors:** Riccardina Tesse, Claudia Paglialunga, Serena Braccio, Lucio Armenio

**Affiliations:** 1 Department of Biomedicine of the Developmental Age, Pediatric Unit "S. Maggiore", University of Bari, Bari, Italy

## Abstract

**Background:**

The therapy for cow's milk proteins allergy (CMPA) consists in eliminating cow's milk proteins (CMP) from the child's diet. Ass's milk (AM) has been recently considered as substitute of CMP. This prospective study investigated tolerance and nutritional adequacy of AM in children with CMPA from Southern Italy.

**Methods:**

Thirty children (aged 6 months to 11 years) with suspected CMPA were enrolled. They underwent skin prick tests and bouble-blind, placebo controlled food challenge to CMP. After confirming the diagnosis of CMPA, patients received fresh AM in a open challenge. Specific serum CMP and AM IgE, and biochemical parameters in blood were also assessed. Auxological evaluations were performed in all subjects at entry (T0) and after 4–6 months (T1) of AM intake.

**Results:**

Twenty-five children resulted elegible for the study, and 24 out of 25 subjects (96%) tolerated AM at the food challenge. Auxological data resulted improved by the end of the study in all patients, while blood biochemical parameters did not vary during the follow-up.

**Conclusion:**

Our data confirm the high rate of AM tolerability in children with moderate symptoms of CMPA. Moreover, we found that AM seems to have nutritional adequacy in subjects with a varied diet.

## Introduction

Cow's milk protein allergy (CMPA) is a common disease occurring in childhood and its prevalence approximates 3% during the first 3 years of life [1]. Clinical manifestations of CMPA are atopic dermatitis (AD), urticaria/angioedema, gastrointestinal symtoms, and less frequently, respiratory disorders, such as wheezing and asthma. The therapy for CMPA consists in eliminating cow's milk proteins (CMP) from the child's diet. Extensively hydrolyzed formulas (eHF) are currently the most used substitutes of CMP. They have a good nutritional value, but are not tolerated by all patients with CMPA, are quite expensive and present poor palatability. Also amino-acid formulas have an unpleasant taste, and should be considered in severe clinical manifestations of food allergy or when eHF are not effective [2]. Soy protein-based formulas have a moderate palatability and provide appropriate nutrition. However there are not reccommended for the tratment of young children with CMPA because of the potential to evoke allergic reactions [3]. For these reasons, other mammalian milks have been considered to replace cow's milk (CM), including ass's milk (AM). The nutritional composition of AM is similar to that of human milk [4,5]. Furthermore, AM has an acceptable taste. There are in literature few clinical Italian studies concerning the allergenicity and tolerability of AM, and their results are not conclusive [6-8]. Recently, Monti et al. have documented that the tolerability of AM was 82.6% in their selected cohort of children with CMPA, without other alternative to the use of common CMP substitutes [6]. Other Authors have emphasized the risk of potential cross-reactivity between CMP and AM proteins, suggesting that more *in vivo *and *in vitro *studies are required to optimize dietary needs for infants with CMPA [9]. Furthermore, some authors have found an impaired growth in infants affected by CMPA, who received different type of CMP substitutes [10]. We aimed to investigate the AM tolerability and nutitional adequacy in children with CMPA from Apulia, a Southern Italy region where AM is readily available and frequently used also in healthy children.

## Methods

Between January and July 2008 we recruited 30 consecutive children who attended our University Department of Pediatric Allergy and Immunology. Inclusion criteria were: i) suspected clinical history of CMPA; ii) absence of concomitant chronic diseases; iii) antihistamine therapy and/or systemic corticosterois not administred in the 4 week preceeding the study. The local Ethics Committee approved the study protocol. Informed consent to partecipate to the study was obtained from the participants or legal guardians. Patients underwent a full history and physical examination at entry. The diagnosis of CMPA was made on the basis of a CMP elimination diet (for 4 weeks), followed by bouble-blind, placebo controlled food challenge (DBPCFC). The DBPCFCs were performed at the clinic, with full facilities for resuscitation available. Fresh CM was administred at increasing doses of 0.1, 0.3, 1.0, 3.0, 10.0, 30.0, and 100 ml, using Neocate^® ^(Nutricia, Liverpool, UK) as placebo. Pear juice was mixed to both CM and placebo in equal parts to mask the taste. The time interval between each dose was 20 min [11]. After completing the DBPCFC, children were kept under observation for at least 6 h and then discharged. Before food challenge, skin prick tests (SPT) were also performed using fresh CM, AM, pear juice and other common food- and aero-allergens, according to a protocol described elsewhere (positive if wheal diameter > 3 mm) [12]. Serum levels of specific IgE (alpha-lactalbumin, beta-lactoglobulin, casein, AM) were determined by the automated Pharmacia CAP System FEIA (Pharmacia Diagnostics AB, Uppsala, Sweden), with a cut-off point for positivity set at 0.35 KU/l.

After confirming the diagnosis of CMPA by DBPCFC, patients received fresh AM (ass-farming of Alberobello, Bari, Italy) in a open challenge, as described for CM. Fresh AM was analyzed for sterility criteria by the Experimental Zooprophylactic Institute of Putignano, Bari, Italy. In case of clinical tolerance, AM was included into the child's diet, which was appropriately balanced depending on age requirements.

The study design included follow-up in the form of clinical check-up and auxological evaluation at entry (T0), and after 4–6 months (T1) of AM consumption.

Standing height (H) was evaluated with a wall-mounted Harpenden Stadiometer. With patients in underwear, weight (W) was measured with an electronic scale with digital readings accurate to 0.1 kg. Body mass index (BMI) was calculated dividing W in kilograms by the square of H in meters, and it was expressed as centiles according to the Italian growth charts [13].

Blood levels of biochemical and metabolic parameters (iron, calcium, proteins, cholesterol, triglycerides, glycaemia, folic acid, etc.) from all subjects, were assessed at each observational time period. Also hidden faecal blood was sought at the beginning and at the end of the study.

### Statistical analysis

Statistical analyses were performed using SPSS^® ^for Windows software (SPSS Inc., version 15.0, Chicago, IL). Data were expressed as the mean ± SD. Mann-Whitney tests were used to compare groups, and correlations between variables were performed using the Sperman rank correlation test. Paired-samples t test was used to compare data before and after AM intake for the group of CMPA patients. Significance was defined as p < 0.05.

## Results

All enrolled children fulfilled the inclusion criteria. Symptoms at first observation were cutaneous (atopic dermatitis, 24/20; urticaria/angioedema, 14/30), gastrointestinal (12/30), and/or respiratory (8/30). Patient's clinical data are summarized in Table 1. Twenty-seven children (90%) were affected by an IgE-mediated form of CMPA, while only two subjects (7%), who presented gastrointestinal manifestations, had a non IgE-mediated CMPA, with negative SPT and CMP-specific IgE. All patients were SPT-negative for pear juice.

**Table 1:** Characteristics of the patients enrolled in the study

**Patient's Characteristics**	
Male/Female ratio	1:1
Age (y), median (range)	4.5 (6.0 mo-10 y)
Total serum IgE at baseline (IU/mL)	335,93
Positive Skin Prick Tests to: (n)	
Cow's milk	27
Ass's milk	2
Wheat	2
Hen's egg	7
Soy	4
Pollens	5
Dust mite	7
Molds	2
Pets with fur	4
	
Positive specific IgE to: (n)	
alpha-lactalbumin	21
beta-lactoglobulin	17
casein	9
Ass's milk	2

The CMPA was confirmed with DBPCFC in 28 children. The parents of one out of two children with SPT positive for AM and the concomitant presence of serum specific IgE for AM refused to give their consent to the ass's milk challenge. In two other cases parents of childs with severe reactions to CM challege did not consent to continue the study. The AM was tolerated by 24 out of 25 children who were tested with the food challenge (22 with IgE-mediated CMPA and 2 with non-IgE-mediated disease). The patient with CMPA and positive AM specific IgE and SPT for AM had a systemic reaction at the food challenge with cutaneous lesions, cought and vomiting.

The auxological evaluations at enrolement (T0) and at the end of the study (T1) are shown in Table 2. All children had an improvement of their W and H at T1. Moreover, at entry all children presented a BMI corresponding to the normalweight (50° centile), which was kept unchanged by the end of the study.

**Table 2:** Auxological parameters of the study population by sex and age at enrolement (T0) and at the end of the study (T1)

**W (Kg) mean ± SD**	**H (cm) mean ± SD**	**BMI mean ± SD** **(centiles)**
**All children**		
T0 17,3 ± 9,6	T0 100,3 ± 14,9	T0 16,1 ± 1,5 (50°)
T1 19,4 ± 11,6	T1 105,4 ± 23,2	T1 16,0 ± 2,1 (50°)
*p = 0.02*	*p < 0.001*	*p = ns*
**Boys**		
T0 16,9 ± 6,7	T0 102,2 ± 23,7	T0 15,8 ± 1,1 (50°)
T1 18,3 ± 7,2	T1 106,3 ± 20,8	T1 15,6 ± 0,8 (50°)
*p < 0.01*	*p = 0.03*	*p = ns*
**Girls**		
T0 17,9 ± 13,3	T0 98,1 ± 29,0	T0 16,5 ± 2,0 (50°)
T1 20,6 ± 16,4	T1 104,2 ± 28,2	T1 16,6 ± 3,0 (50°)
*p = ns*	*p < 0.01*	*p = ns*

*p significance for *Paired T-test*

*ns *= not significant

Any relevant variation of blood biochemical and metabolic parameters was observed (data not shown), and also the faecal tests resulted negative.

## Discussion

In this study we report an experience on clinical tolerability of AM, which was high at the food challenge (24/25, 96%), made in an Italian Region, Apulia, where AM is readily available and frequently used because of the presence of several ass's farming. AM was tolerated by our patients either with the IgE- and the non-IgE-mediated CMPA. All enrolled subjects found it acceptable due to its palatability, and did not interrupted the study. AM has been considered an alternative to CM considering that its protein composition is similar to human milk [14]. However, it is a low-calory food and thus in our study we enrolled children older than 6 months who did not have an exclusive milk diet. It is noteworthy to highlight the fact that patients with severe reaction to CM refused to continue this study, thus our results are referred to subjects with a mild to moderate form of CMPA. Only one recruited patient had a systemic food reaction during the AM challenge. He was the only tested child who had SPT positive for AM and high serum levels of AM specific IgE, suggesting the predictive positive value of these tests also for AM allergy.

Other Italian authors have documented the efficacy of this food in treating children with CMPA. Vita et al. showed that the rate of tolerability of AM in patients with AD and CMPA was 88%, and that AM improved child's eczema [7]. Similarly, Monti et al. assessed the tolerance of AM (82.6%) in a selected population of children with CMPA, for whom it was not possible to use any other cow's milk substitute [6]. However, it must be taken into account the potential cross-reactivity of AM proteins with CMP, also suggested by the above mentioned studies that reported some severe reactions to AM in their study cohorts [6-9].

Furthermore, our data show an adequate increase in weight and length/stature in all recruited children. Their BMI centiles measured after 4–6 months of AM administration were stable compared to those at the beginning of the study. Similar observations were reported by Monti in the early months of AM intake [6]. These Authors concluded that the effect on growth of AM is related to its ability to fill some nutritional gaps present in the diet of the subjects treated. Other authors have found an impaired growth in infants affected by CMPA, who received different type of CMP substitutes [10]. We suggest that a longer follow-up study is needed in order to reach reliable results. We also verified that patients' biochemical and metabolic parameters in the blood did not vary during the study period. Taken toghether these results suggest that AM might be considered nutritionally adequate in children with a varied diet.

## Competing interests

The authors declare that they have no competing interests.

## Authors' contributions

TR partecipated in the study design and interpretations of results, and performed statistical analysis and the preparation of the manuscript. PC partecipated in the data collection and helped to draft the manuscript. BS has provided contribution to the data collection. AL conceived the study, partecipated in its design and coordination and helped in the interpretation of results. All authors read and approved the final manuscript.
